# Recessive *LAMA5* Variants Associated With Partial Epilepsy and Spasms in Infancy

**DOI:** 10.3389/fnmol.2022.825390

**Published:** 2022-05-19

**Authors:** Sheng Luo, Zhi-Gang Liu, Juan Wang, Jun-Xia Luo, Xing-Guang Ye, Xin Li, Qiong-Xiang Zhai, Xiao-Rong Liu, Jie Wang, Liang-Di Gao, Fu-Li Liu, Zi-Long Ye, Huan Li, Zai-Fen Gao, Qing-Hui Guo, Bing-Mei Li, Yong-Hong Yi, Wei-Ping Liao

**Affiliations:** ^1^Key Laboratory of Neurogenetics and Channelopathies of Guangdong Province and the Ministry of Education of China, Department of Neurology, Institute of Neuroscience, Second Affiliated Hospital of Guangzhou Medical University, Guangzhou, China; ^2^The Second School of Clinical Medicine, Southern Medical University, Guangzhou, China; ^3^Department of Pediatrics, Affiliated Foshan Maternity & Child Healthcare Hospital, Southern Medical University, Foshan, China; ^4^Epilepsy Center, Qilu Children’s Hospital of Shandong University, Jinan, China; ^5^Department of Pediatrics, The Second Hospital, Cheeloo College of Medicine, Shandong University, Jinan, China; ^6^Department of Neurology, Guangdong General Hospital, Guangdong Academy of Medical Sciences, Guangzhou, China; ^7^Department of Neurology, The First People’s Hospital of Foshan, Foshan, China

**Keywords:** *LAMA5* gene, infant-onset epilepsy, laminins, trios-based WES, spasms

## Abstract

**Objective:**

The *LAMA5* gene encodes the laminin subunit α5, the most abundant laminin α subunit in the human brain. It forms heterotrimers with the subunit β1/β2 and γ1/γ3 and regulates neurodevelopmental processes. Genes encoding subunits of the laminin heterotrimers containing subunit α5 have been reported to be associated with human diseases. Among *LAMAs* encoding the laminin α subunit, *LAMA1-4* have also been reported to be associated with human disease. In this study, we investigated the association between *LAMA5* and epilepsy.

**Methods:**

Trios-based whole-exome sequencing was performed in a cohort of 118 infants suffering from focal seizures with or without spasms. Protein modeling was used to assess the damaging effects of variations. The *LAMAs* expression was analyzed with data from the GTEX and VarCards databases.

**Results:**

Six pairs of compound heterozygous missense variants in *LAMA5* were identified in six unrelated patients. All affected individuals suffered from focal seizures with mild developmental delay, and three patients presented also spasms. These variants had no or low allele frequencies in controls and presented statistically higher frequency in the case cohort than in controls. The recessive burden analysis showed that recessive *LAMA5* variants identified in this cohort were significantly more than the expected number in the East Asian population. Protein modeling showed that at least one variant in each pair of biallelic variants affected hydrogen bonds with surrounding amino acids. Among the biallelic variants in cases with only focal seizures, two variants of each pair were located in different structural domains or domains/links, whereas in the cases with spasms, the biallelic variants were constituted by two variants in the identical functional domains or both with hydrogen bond changes.

**Conclusion:**

Recessive *LAMA5* variants were potentially associated with infant epilepsy. The establishment of the association between *LAMA5* and epilepsy will facilitate the genetic diagnosis and management in patients with infant epilepsy.

## Introduction

The *LAMA5* gene (OMIM* 601033) encodes the laminin subunit α5, expressed in the human brain, especially in the cortex and during the early stages of life (Durkin et al., 1997). The laminin subunit α5 forms heterotrimers with subunits β1/β2 and γ1/γ3 and regulates neurodevelopmental biological processes, including epiblast polarization, neurite outgrowth, neuronal migration, synaptic stability, and cell adhesion, differentiation, migration, and signaling (Liang and Crutcher, 1992; Luckenbill-Edds, 1997; Domogatskaya et al., 2012; Mukherjee et al., 2020). In mice, homozygous knock-out of *LAMA5* caused lethality throughout fetal growth and development, and led to exencephaly, megalencephaly, and other neural tube defects (Miner et al., 1998; Kikkawa and Miner, 2006). Genes encoding subunits of the laminin heterotrimers containing subunit α5 have been reported to be associated with human diseases. Among *LAMAs* encoding the laminin α subunit, *LAMA1-4* have also been reported to be associated with human disease. The association between the *LAMA5* gene, which encodes the most abundant laminin α subunit in the human brain, and human diseases, has not been determined.

Epilepsy is one of the most common neurological disorders in children with an estimated prevalence of 4–5 per 10,000. Infancy is the critical period of brain development, and epilepsy presents the highest incidence in infancy (Wirrell et al., 2011; Eltze et al., 2013; Hirose, 2013). Multiple seizures may appear in infancy epilepsy, such as focal (partial), myoclonic, spasms seizures, and spasms typically occur in infancy (Bayat et al., 2021). Clinically, both spasms and focal seizures are common in infancy. A proportion of infants with epilepsy have acquired causes, such as trauma, infection, and immune, but the etiologies in the majority are unknown. Previous studies have shown that genetic factors play an important role in the etiology of infant epilepsy (Gan et al., 2019; Kang et al., 2019). The established causative genes include *PRRT2, KCNQ2, SCN1A, SCN2A, STXBP1, CDKL5*, and *ARX*, which contribute to approximately 19% of patients with infant-onset epilepsy (Song et al., 2021).

In this study, trios-based whole-exome sequencing (WES) was performed in a cohort of infants with epilepsy. Six pairs of compound heterozygous missense variants in *LAMA5* were detected in six unrelated cases. The present study suggests that recessive *LAMA5* variants were potentially associated with infant epilepsy.

## Materials and Methods

### Subjects

One hundred and eighteen infants who suffered from focal seizures without any acquired causes were recruited from four hospitals, including the Second Affiliated Hospital of Guangzhou Medical University, Foshan Maternal and Child Health Hospital, the Second Affiliated Hospital of Shandong University, and Children’s Hospital of Shandong University, from June 2019 to July 2021. Clinical information of the affected individuals was collected, including age at onset, type and frequency of seizures, family history, systemic and neurological findings, and effective antiepileptic drugs. The structural abnormalities were detected by brain magnetic resonance imaging (MRI) scans. Long-term (24 h) video electroencephalography (EEG) monitoring records were performed with electrodes being arranged according to the international standard of 10–20 reduced montage system. The procedures of open-close eyes test, hyperventilation, intermittent photic stimulation, and sleeping recording were obtained. The EEG results were reviewed by at least two qualified electroencephalographers. The Chinese version of the Gesell development scales was utilized in the neurodevelopment evaluation of the participants according to their ages. Its scores were evaluated using tests for gross motor, fine motor, adaptive behavior, language, and personal-social behavior. Epileptic seizures and epilepsy syndromes were diagnosed according to the criteria of the Commission on Classification and Terminology of the International League Against Epilepsy (1981, 1989, 2001, 2010, and 2017). All patients were followed up for more than 1 year.

For the controls, WES was performed on 296 healthy Chinese volunteers who served as a normal control group as our previous report (Wang et al., 2018). Frequencies of the identified variants were also compared with that in the other control populations, including East Asian and general populations in the Genome Aggregation Database (gnomAD^1^), and the 8,364 persons without known neuropsychiatric conditions in the Epi25 WES Browser^2^ (Epi25 Collaborative, 2019; Karczewski et al., 2020).

This study adhered to the principles of the International Committee of Medical Journal Editors concerning patient consent for research or participation and received approval from the Ethics Committee of the Second Affiliated Hospital of Guangzhou Medical University. Written informed consents were provided by the patient’s legal guardians.

### Whole Exon Sequencing

Blood samples of the probands, their parents, and other available family members were collected to identify the source of the genetic variation and to aid in the analysis of the pathogenicity of variants. Genomic DNAs were extracted from blood samples using the Qiagen Flexi Gene DNA kit (Qiagen, Hilden, Germany). WES was performed using a NextSeq500 sequencing instrument (Illumina, San Diego, CA, United States) following the standard procedures previously described (Wang et al., 2018). The sequencing data were generated by massively parallel sequencing with an average depth of >125× and >98% coverage of the capture region on the chip for obtaining high-quality reads that were mapped to the Genome Reference Consortium Human genome build 37 by Burrows–Wheeler alignment. Single-nucleotide point variants and indels were called with the Genome Analysis Toolkit.

### Genetic Analysis

A case-by-case analytical approach was adopted to identify candidate causative variants in each trio. Primarily, the rare variants were prioritized with a minor allele frequency <0.005 in the Genome Aggregation Database (see Text Footnote 1). Then, potentially pathogenic variants were retained, including frameshift, nonsense, canonical splice site, initiation codon, and missense variants predicted as being damaging *in silico* tools. Lastly and importantly, the potentially disease-causing variants in each trio were analyzed with an individualized protocol. The variants in each trio were sorted according to the following five models: (1) epilepsy-associated gene model (Wang et al., 2017); (2) *de novo* variant dominant model; (3) autosomal recessive inheritance model, including homozygous and compound heterozygous variants; (4) X-linked model; (5) co-segregation analysis model. To identify novel epilepsy-associated genes, the known epilepsy-associated genes were put aside. Genes with *de novo* variants, bi-allelic variants, hemizygous variants, or variants with segregations, which represent the genetic difference between the patients and normal individuals in a family and potentially explain the occurrence of disease, were selected for further studies to define the gene-disease association. *LAMA5* emerged as one of the candidate genes with recurrent bi-allelic variants in this cohort of infancy epilepsy. Sanger sequencing was used to validate the candidate variants. All *LAMA5* variants identified in this study were annotated into the reference transcript NM_005560.4.

### Bioinformatic Analyses

Protein modeling was performed by using the Iterative Threading ASSEmbly Refinement software (I-TASSER^3^) to evaluate the damaging effect of candidate variants (Yang and Zhang, 2015). The confidence of each model was quantitatively measured by a *C*-score in the range of [−5, 2]. PyMOL Molecular Graphics System (Version 2.3.2; Schrödinger, LLC; New York, NY, United States) was used for three-dimensional protein structure visualization and analysis. I-Mutant Suite server was used for the prediction of protein stability changes upon single-nucleotide variants that lead to changes in the amino acid^4^ (Capriotti et al., 2005). The alteration of the protein stability was assessed by the free energy change value (ΔΔG, kcal/mol). Values greater than 0.5 kacl/mol imply a large increase in protein stability, values less than −0.5 kacl/mol are considered to be a large decrease in protein stability, and others imply neutral stability. The consequences of all the missense variants were predicted by several common tools, including SIFT, PolyPhen2_HVAR, CADD, MutationTaster, GenoCanyon, fitCons, M_CAP, and GERP.

### Statistical Analysis

R (version 4.0.3) was used for data processing. The frequencies of the *LAMA5* variants between the epilepsy cohort and the controls were compared by a two-sided Fisher exact test (CONVERGE Consortium, 2015). The burden of recessive variants was analyzed according to the method recommended recently (Martin et al., 2018). *P*-value < 0.05 was considered statistically significant.

## Results

### Identification of *LAMA5* Variants

**FIGURE 1:**
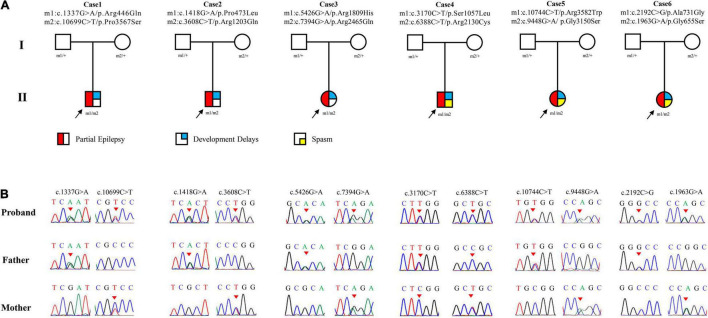
Genetic data of cases with *LAMA5* variants. **(A)** Pedigrees of the six cases with *LAMA5* mutations and their corresponding phenotypes. **(B)** DNA sequence chromatogram of the *LAMA5* mutations. Arrows indicate the positions of the variants.

Six pairs of compound heterozygous missense variants, i.e., (c.1337G > A/p.Arg446Gln and c.10699C > T/p.Pro3567Ser), (c.1418G > A/p.Pro473Leu and c.3608C > T/p.Arg1203Gln), (c.5426G > A/p.Arg1809His and c.7394G > A/p.Arg2465Gln), (c.3170C > T/p.Ser1057Leu and c.6388C > T/p.Arg2130Cys), (c.9448G > A/p.Gly3150Ser and c.10744C > T/p.Arg3582Trp), and (c.1963G > A/p.Gly655Ser and c.2192C > G/p.Ala731Gly), were identified in six unrelated cases with focal seizures, including three infants with also spasms (Figures 1A,B and Table 1). The compound heterozygous variants originated from their asymptomatic mothers and fathers, consistent with a classical recessive inheritance pattern.

**TABLE 1:** Clinical features of the patients with *LAMA5* variants.

	Variants (NM_005560.4)	Sex	Age	Seizure onset	Seizure course	Seizure timing	Seizure-free duration	AEDs	EEG	Brain MRI	Development delay
Case 1	c.1337G > A/p.Arg446Gln c.10699C > T/p.Pro3567Ser	M	4 yr.	9 mo.	sGTCS ∼7/yr. FS once in 3 yrs.	Nocturnal mostly	3 yr	LEV, VPA	Spike-slow waves in middle and posterior temporal areas.	Normal	Mild
Case 2	c.1418G > A/p.Pro473Leu c.3608C > T/p.Arg1203Gln	M	4 yr.	5 mo.	sGTCS 1-2 time/wk.	Diurnal and nocturnal	3 yr	VPA	Sharp and spike waves in frontal, central, and pretemporal areas.	Normal	Mild
Case 3	c.5426G > A/p.Arg1809His c.7394G > A/p.Arg2465Gln	F	4 yr.	1 mo.	1–2 time/wk.	Diurnal and nocturnal	4 yr	VPA	Spikes in the frontal and central areas.	Normal	Mild
Case 4	c.3170C > T/p.Ser1057Leu c.6388C > T/p.Arg2130Cys	M	1.5 yr.	3 mo.	Spasms and CPS, 1–3 times/days	Diurnal mostly	1 yr	TPM, ATCH	Spike-slow waves in the anterior area and generalized spikes and spike-slow waves.	Normal	Mild
Case 5	c.9448G > A/p.Gly3150Ser c.10744C > T/p.Arg3582Trp	F	1.5 yr.	5 mo.	Spasms and CPS, 1–2 times/day.	Nocturnal mostly	1 yr	TPM, ATCH	Spike-slow and sharp-slow waves in the bilateral posterior area and generalized spikes and spike-slow waves.	Normal	Mild
Case 6	c.2192C > G/p.Ala731Gly c.1963G > A/p.Gly655Ser	F	2.5 yr.	3 mo.	Spasms and CPS, 2–3 times/day.	Diurnal and nocturnal	–	CNZ, LEV, TPM, LTG	Spikes and spike-slow waves in bilateral temporal, frontal and central areas.	Normal	Mild

*AEDs, antiepileptic drug; ATCH, adrenocorticotropic hormone; CPS, complex partial seizure; EEG, electroencephalogram; F, female; FS, febrile seizure; LEV, levetiracetam; LTG, lamotrigine; M, male; mo, month; MRI, magnetic resonance imaging; sGTCS, secondary generalized tonic-clonic seizure; TPM, topiramate; VPA, valproate; wk, week; yr, year.*

These variants presented no or low frequencies (MAF < 0.005) in the gnomAD databases (Table 2). Nine of the variants did not present in the normal control of Epi25 WES Brower, and the other three variants presented extremely low frequencies (MAF < 0.0005). None of the variants, except p.Ala731Gly, were presented in the 296 normal controls.

**TABLE 2:** Analysis of the aggregate frequency of *LAMA5* variants identified in this study.

Variants (NM_005560.4)	Allele count/number in this study	Allele count/number in the six controls						Homozygotes in the controls of gnomAD
			
		The controls of 296 healthy volunteers	The controls of Epi25 WES Brower	GnomAD-all population	The controls of gnomAD-all population	GnomAD-East Asian	The controls of gnomAD-East Asian	
c.1337C > T/p.Arg446Gln	1/236	−/−	1/16864	6/280278	1/109070	2/19918	0/9034	0
c.1418G > A/p.Pro473Leu	1/236	−/−	3/16870	15/263638	2/100060	0/19346	0/8626	0
c.1963G > A/p.Gly655Ser	1/236	−/−	−/−	7/248058	2/108800	0/18250	0/8948	0
c.2192C > G/p.Ala731Gly	1/236	1/592	−/−	14/281176	6/109254	14/19938	6/9046	0
c.3170C > T/p.Ser1057Leu	1/236	−/−	−/−	−/−	−/−	−/−	−/−	–
c.3608G > A/p.Arg1203Gln	1/236	−/−	−/−	28/270370	8/104670	7/19516	1/8836	0
c.5426G > A/p.Arg1809His	1/236	−/−	2/16860	1/251170	1/109360	0/18378	0/9038	0
c.6388C > T/p.Arg2130Cys	1/236	−/−	−/−	125/261646	65/110372	10/19184	6/9426	0
c.7394G > A/p.Arg2465Gln	1/236	−/−	−/−	108/253406	50/108836	78/19018	35/9738	0
c.9448G > A/p.Gly3150Ser	1/236	−/−	−/−	108/272790	56/117014	17/19536	7/9698	0
c.10699C > T/p.Pro3567Ser	1/236	−/−	−/−	1/248280	−/−	1/18254	−/−	–
c.10744C > T/Arg3582Trp	1/236	−/−	0/16844	59/277032	27/118346	38/19806	16/9870	0
Total	12/236(5.08 × 10^–2^)	1/592(1.69 × 10^–3^)	6/16844(3.56 × 10^–4^)	472/248058(1.9 × 10^–3^)	218/100060(2.18 × 10^–3^)	167/18250(9.15 × 10^–3^)	71/8626(8.04 × 10^–3^)	0
*P*-value		2.243 × 10^–6^	<2.2 × 10^–16^	8.306 × 10^–14^	4.728 × 10^–13^	3.36 × 10^–6^	1.749 × 10^–6^	–
OR (95% CI)		31.589(4.628–1348.630)	149.545(51.506–484.264)	28.104(14.202–50.423)	24.538(12.299–44.594)	5.799(2.894–10.599)	6.452(3.139–12.204)	–

*P-values and odds ratio were estimated with 2-sided Fisher’s exact test.*

*CI, confidence interval; gnomAD, Genome Aggregation Database; OR, odd ratio; WES, whole-exome sequencing.*

When the burden of recessive variants was analyzed, (Martin et al., 2018) the *LAMA5* variants in the present cohort were significantly more than the expected number by chance in the East Asian population (MAF < 0.005, *P* = 6.4654 × 10^–6^). Furthermore, the aggregate frequency of the variants in this cohort was significantly higher than that in the six controls (Table 2), including the controls of 296 normal individuals (12/236 vs. 1/592, *p* = 2.243 × 10^–6^), the normal control of Epi25 WES Browser (vs. 6/16844, *p* < 2.2 × 10^–16^), the gnomAD-all population (vs. 472/248,058; *p* = 8.306 × 10^–14^), the controls of gnomAD-all population (vs. 218/100,060; *p* = 4.728 × 10^–13^), the gnomAD-East Asian population (vs. 167/18250, *p* = 3.36 × 10^–6^), and the controls of the gnomAD-East Asian population (vs. 71/8836, *p* = 1.749 × 10^–6^).

All *LAMA5* variants identified in this study were predicted to be damaging by at least two *in silico* tools (Supplementary Table 1). The probability that transcript falls into distribution of recessive genes (pRec) is 0.99353 for the *LAMA5* gene, indicating that it is very likely intolerant to recessive loss-of-function variations (Karczewski et al., 2020). None of the six affected individuals had pathogenic or likely pathogenic variants in genes known to be associated with epilepsy (Wang et al., 2017).

### Clinical Features of the Cases With *LAMA5* Variants

**FIGURE 2:**
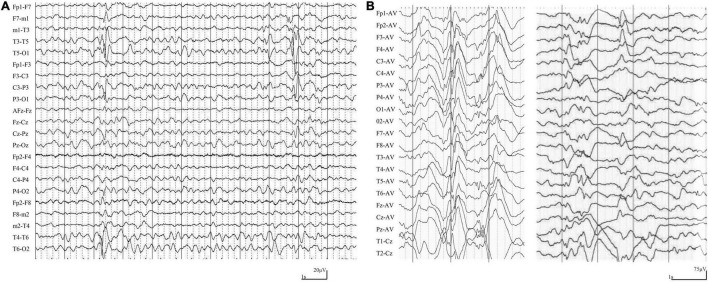
Ictal and interictal EEG in the cases with *LAMA5* variants. **(A)** Interictal EEG in case 1 at the age of 8 months showed irregular spike-slow waves in the middle and posterior temporal region. **(B)** Interictal EEG in case 4 at the age of 3 months showed diffuse high amplitude spike and spike-slow waves, and focal spike-slow waves in the anterior region.

The main clinical features of the six patients with *LAMA5* variants were summarized in Table 1. All patients showed infant-onset epilepsy with onset ages ranging from 1 to 9 months. The patients of cases 1, case 2, and case 3 suffered from infrequent focal seizures or secondary tonic-clonic seizures (monthly or yearly) and became seizure-free after treatment with valproate monotherapy or valproate in combination with levetiracetam. Their EEGs showed bilateral, unilateral, and multiple discharges, predominantly at the frontal, central, and temporal lobe, mainly during sleep (Figure 2A). The patients of case 4 and case 5 had daily focal seizures with spasms. Seizure-free was achieved after treatment with a combination of adrenocorticotropic hormones and topiramate. The patient of case 6 also had focal seizures and spasms (daily). The spasms and focal seizures disappeared after treatment, but myoclonic seizures appeared, which were infrequent (3–4 times/month). Interictal multifocal and generalized discharges were recorded in the three cases with spasms (Figure 2B).

These patients were full-term and delivered without abnormalities. Brain MRI was normal in the six cases. All patients showed mild global developmental delays.

### Molecular Alteration of Laminin Subunit α5

**FIGURE 3:**
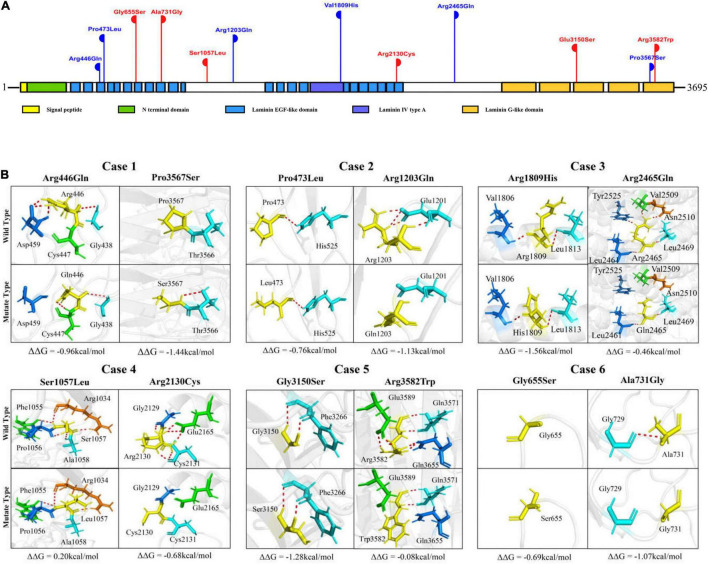
Schematic illustration of *LAMA5* variants. **(A)** Schematic illustration of the laminin subunit α5 and the location of the *LAMA5* variants identified in this study. Two variants of the same height represent a pair of biallelic mutations. The variants of red-colored represent cases with both spasms and focal seizures; those of blue-colored represent cases with only focal seizures. **(B)** Hydrogen bond changes and free energy stability changes (ΔΔ*G*, Kcal/mol) value of the variants from the present study.

The laminin subunit α5 contains one signal peptide, one Laminin N terminal domain, 22 Laminin EGF-like domains, one Laminin IV type A domain, and five Laminin G-like domains (Uniport-id: O15230) (Figure 3A). Five of the variants were located in Laminin EGF-like domains, including p.Arg446Gln, p.Pro473Leu, p.Gly655Ser, p.Ala731Gly, and p.Arg2130Cys. Three variants, p.Glu3150Ser, p.Pro3567Ser, and p.Arg3582Trp, were located in the Laminin G-like domains. Variant p.Val1809His was located in the Laminin IV type A domain. The other three variants p.Ser1057Leu, p.Arg1203Gln, and p.Arg2465Gln are located between structural domains (Figure 3A).

The molecular effects of the variants were analyzed by using I-TASSER for protein modeling and PyMOL for visualization. Eight of the variants, including p.Arg446Gln, p.Pro3567Ser, p.Arg1203Gln, p.Arg2465Gln, p.Ser1057Leu, p.Arg2130Cys, p.Arg3582Trp, and p.Ala731Gly changed their hydrogen bonds with surrounding residues. The other four variants, p.Pro473Leu, p.Gly655Ser, p.Arg1809His, and p.Gly3150Ser, did not change their hydrogen bonds but were predicted to decrease the protein stability significantly with ΔΔ*G* values less than −0.5 kcal/mol. In each pair of compound heterozygous variants, at least one variant had hydrogen bonds change (Figure 3B).

It is notable that among the compound heterozygous variants in the three cases with only focal seizures (case 1, case 2, and case 3), two variants of each pair were located in different structural domains or domains/links (Figure 3A). Case 1 had the compound heterozygous variants with two variants located furthest apart (p.Arg446Gln and p.Pro3567Ser); the patient showed a milder phenotype than the others, e.g., the latest onset age (8-month-old) and infrequent seizure frequency (yearly). In the three cases with spasms, two pairs of compound heterozygous variants (case 5 and case 6) were constituted by two variants in the identical functional domains. The two variants of case 4 were in non-identical structural domains but presented the most pronounced hydrogen bonding changes (a total of eight hydrogen bonds disrupted).

### The Expression Profile of *LAMAs*

**FIGURE 4:**
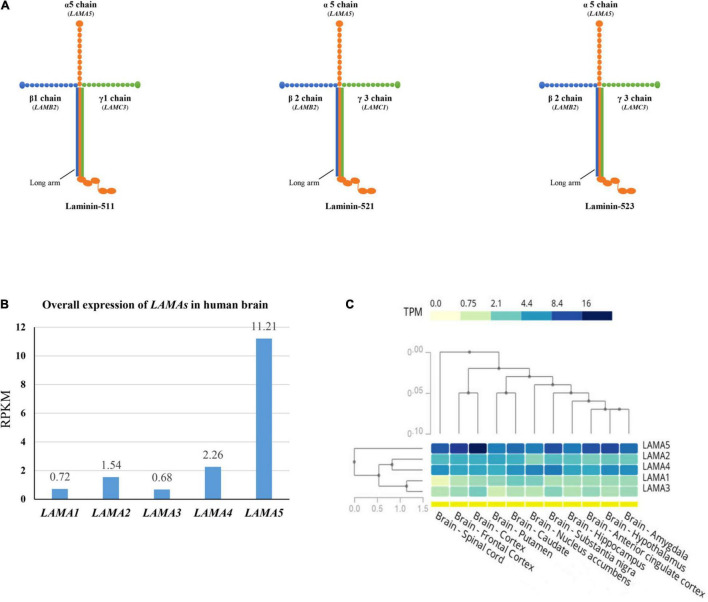
Schematic illustration of laminin heterotrimers and the expression profile of *LAMAs*. **(A)** Schematic illustration of the heterotrimers containing laminin subunit α5. Laminin subunit α5 formed laminin-511 with subunit β1 and γ1, laminin-521 with subunit β2 and γ1, and laminin-523 with subunit β2 and γ3. **(B)** The overall expression of *LAMAs* in the human brain. RPKM, Reads Per Kilobase per Million mapped reads. **(C)** Heatmap and hierarchical clustering of *LAMAs* expression in the sub-regions of human brain. Columns represent individual sub-region and rows represent individual genes. The darker the color, the higher the expression. The lines represent cluster analysis. More details were presented in the GTEx database (www.gtexportal.org).

The functional laminins are cruciform heterotrimers that consist of three short arms formed by the N-terminal portion of α, β, and γ subunits, respectively, and a long arm polymerized by the C-terminal parts of the three subunits (Figure 4A). The laminin short arms (N-terminus) are involved in laminin’s ability to polymerize with those of other laminins to form a polygonal network, while the laminin long arm interacts with the extracellular matrix components to regulate biological processes (Hohenester, 2019). Laminin subunit α5 appears in laminin-511 (with β1 and γ1), laminin-521 (with β2 and γ1), and laminin-523 (with β2 and γ3). Previous studies show that laminins containing subunit α5 play an essential role in embryonic development and are intensely expressed on the surface of the ectoderm (Copp et al., 2011). Tissue-specific expression is the basis of gene function and subsequently the clinical phenotype. We thus compared the expression of *LAMAs* in the human brain from the data in VarCards and Genotype-Tissue Expression databases (GTEx Consortium, 2013). The *LAMA5* gene presented the highest expression in the human brain (Figure 4B). Furthermore, it was more abundant in the cerebral cortex, substantia nigra, frontal cortex, hippocampus, and anterior cingulate cortex (Figure 4C).

## Discussion

The *LAMA5* gene, predominantly expressed in the early stage of life, plays a vital role in neurodevelopmental biological processes such as neurite outgrowth, epiblast polarization, neuronal migration, synaptic stability, and cell adhesion, differentiation, migration, and signaling. A functional study suggested that the laminin subunit α5 was deposited at synapses in the brain and was essential for dendritic spine structural regulation and synapse stability (Omar et al., 2017). Experimentally, mutant protein of *LAMA5* resulted in decreased binding to the synaptic vesicle protein encoded by *SV2A* (Maselli et al., 2018). Abnormalities in the central nervous system were monitored in the conditional allele knockout *LAMA5* mice (Omar et al., 2017). In this study, six pairs of biallelic variants in *LAMA5* were identified in patients with infant-onset epilepsy. All variants presented no or low allele frequencies in controls. The aggregate frequency of the *LAMA5* variants identified in the case cohort was significantly higher than that in controls. The recessive burden analysis also showed that the *LAMA5* variants in the present cohort were significantly more than the expected number in the East Asian population. These findings suggest a potential association between *LAMA5* variants and epilepsy.

Previously, a *de novo* canonical splice site variant (c.10828 + 1G > A) was identified in a patient with developmental delays and epilepsy (Han et al., 2018). In a patient with cortical developmental malformations, a pair of compound heterozygous variants (p.Glu3567Lys and p.Asp2372Asn) were identified (Wiszniewski et al., 2018). One pair of homozygous variants in *LAMA5* (p.Arg2659Trp) and one homozygous variant in *LAMA1* were identified in a patient with the presynaptic congenital myasthenic syndrome who has non-classified facial tics or tics (Maselli et al., 2018). A heterozygous variant in *LAMA5* (p.Val3140Met) was identified in a family with a complex multisystem syndrome, in which several family members with the *LAMA5* variant had seizures (Sampaolo et al., 2017). These data provided clues for the possible association between *LAMA5* and epilepsy, but the pathogenic role of *LAMA5* variants in epilepsy could not be determined due to the variable phenotype, co-appearance of variants in other potentially pathogenic genes, or the single affected case. In the present study, we identified *LAMA5* variants in six unrelated cases, and the other possible pathogenic genes were excluded. Therefore, this study provided direct evidence in supporting the association between *LAMA5* and epilepsy.

In animals, the homozygous *LAMA5* knockout mice model showed preweaning lethality with complete penetrance^5^, while the heterozygous knockout mice showed fewer abnormalities, suggesting a dose-effect. The pRec, a metric for intolerant of biallelic loss-of-function variants, is 0.99353 for the *LAMA5* gene, indicating that it is highly intolerant for recessive loss-of-function variations (Karczewski et al., 2020). The *LAMA5* variants identified in the present study were all biallelic variants. It was noted that among the compound heterozygous variants in the three cases with only focal seizures, two variants of each pair variant were located in different structural domains or domains/links. In the three cases with spasms, two pairs of variants were constituted by two variants in the identical functional domains, and another pair of variants presented the most pronounced hydrogen bonding changes. Taken together that homozygous *LAMA5* knockout mice displayed preweaning lethality with complete penetrance, it was considered that homozygous variants in *LAMA5* would cause the most severe damage effect and resulted in the most severe phenotype or even early death; the compound heterozygous variants with two variants located in identical functional domains potentially created relatively severe damage effect and caused severe epilepsy such as spasms; and the compound heterozygous variants with two variants located in non-identical functional domains potentially caused relatively mild damage effects and led to mild phenotypes. It is suggested that the location of variants in compound heterozygous variants was potentially associated with the phenotype severity, providing one of the explanations for the phenotype variation.

Structurally, the laminin subunit α5 forms heterotrimers with laminin subunits β1/β2 and γ1/γ3, which were encoded by *LAMB1*, *LAMB2*, *LAMC1*, and *LAMC3* that were associated with neurodevelopmental diseases and epilepsy. The *LAMB1* gene is the responsible gene of lissencephaly 5 (OMIM #615191) characterized by focal and spasmodic seizures and psychomotor development delay (Radmanesh et al., 2013; Tonduti et al., 2015). The *LAMB2* gene is the causative gene of Pierson syndrome (OMIM #609049), with which the majority of patients died early and the survivors presented severe neurodevelopmental delays (Pierson et al., 1963; Zenker et al., 2004). The *LAMC1* gene has been repeatedly reported in Dandy–Walker malformation with occipital cephalocele, and most of the affected individuals had an infant-onset intellectual disability with or without seizures (Carvalho et al., 2006; Darbro et al., 2013). Variants in *LAMC3* were the causes of occipital cortical malformations (OMIM #614115), and the affected individuals experienced seizures (Barak et al., 2011). Experimentally, these genes caused neurological abnormalities through a common mechanism of disrupted function of laminin heterotrimers in regulating neuronal migration and other biological processes (Pierson et al., 1963; Zenker et al., 2004; Carvalho et al., 2006; Darbro et al., 2013; Radmanesh et al., 2013; Tonduti et al., 2015). However, the *LAMA5* gene, which encodes one of the indispensable parts of the heterotrimers, has not been confirmed to be associated with neurological disorders. In this study, the patients with biallelic *LAMA5* variants presented focal seizures and developmental delays, suggesting a potential role of *LAMA5* in epilepsy with the involvement of neurodevelopment.

Regarding *LAMA*s that encode the laminin α subunits, the associations between *LAMA1-4* and human diseases have been established. Variants in *LAMA1* cause Poretti–Boltshauser syndrome (OMIM #615960), characterized by delayed motor development, speech delay, and cognitive function; and seizures, tics, and spasticity have also been observed (Aldinger et al., 2014; Elmas et al., 2020). *LAMA2* is the causative gene of autosomal recessive limb-girdle muscular dystrophy-23 (OMIM #618138) and congenital merosin deficient or partially deficient muscular dystrophy (OMIM #607855), in which epilepsy was regarded as one of the core features (Chan et al., 2014; Xiong et al., 2015; Salvati et al., 2021). The *LAMA3* gene is the responsible gene of Herlitz type junctional epidermolysis bullosa (OMIM #226700), generalized atrophic benign epidermolysis bullosa (OMIM #226650), and laryngo-onycho-cutaneous syndrome (OMIM #245660) (Kivirikko et al., 1995; McGrath et al., 1995; Vidal et al., 1995; Nakano et al., 2002; McLean et al., 2003). The *LAMA4* gene is the causative gene of dilated cardiomyopathy 1JJ (OMIM #615235) (Knoll et al., 2007). Compared with the other *LAMA*s genes, the *LAMA5* gene had the highest expression in the human brain and was abundant in the cerebral cortex, substantia nigra, frontal cortex, hippocampus, and anterior cingulate cortex. Generally, tissue-specific expression is the basis of gene function and subsequently the clinical phenotype. The highest expression of *LAMA5* in the human brain provided an anatomical basis for the association between *LAMA5* and neurological diseases.

The proteins encoded by *LAMAs* are of tissue-specific expression and independent function, while the protein encoded by *LAMA5* interacts with that encoded by *LAMBs* and *LAMCs*. Therefore, the possibility of digenic or polygenic mechanisms could not be excluded. No pathogenetic or likely pathogenetic variants in *LAMBs*, *LAMCs*, or other epilepsy genes were identified in these patients, suggesting a potential monogenic role of *LAMA5*. However, the patients with *LAMA5* variants presented a relatively moderate phenotype, and several variants presented in the population with low frequency. Thus, a modifier role of *LAMA5* could not be excluded. Further studies with large cohorts are needed to validate the pathological nature of *LAMA5* variants.

Among the disease-causing genes in humans (OMIM^6^), 1,008 genes were associated with disease in a dominant inheritance pattern, whereas 1,936 genes were in a recessive inheritance pattern. Considering that the genome in humans is diploid, it is possible that recessive variants were more common than dominant variants in the etiology of human diseases. Currently, most infantile spasms-related genes (36/47) (Bayat et al., 2021) and all focal epilepsy-associated genes were of autosomal dominant inheritance. Thus, more attention should be paid to recessive variants in epilepsy. This study revealed *LAMA5* as a potential novel autosomal recessive gene in infant epilepsy, enriching the genetic etiology of epilepsy.

This study has several limitations. The direct functional effects of the variants were not examined. The consequences of the variants on the interactions of the alpha subunit with its partners warrant further studies. Besides point variants, whether CNV in the *LAMA5* gene was pathogenic should also be considered.

## Conclusion

The association between *LAMA5* and epilepsy was supported by multiple pieces of evidence, such as common clinical features, unique gene functions, and statistical evidence. The establishment of the association between *LAMA5* and epilepsy will facilitate the genetic diagnosis and management in patients with infant epilepsy.

## Data Availability Statement

The datasets presented in this study can be found in online repositories. The names of the repository/repositories and accession number(s) can be found below: NCBI GenBank: OM994299 – OM994307 and OM994308 – OM994334.

## Ethics Statement

This study adhered to the principles of the International Committee of Medical Journal Editors concerning patient consent for research or participation and received approval from the Ethics Committee of the Second Affiliated Hospital of Guangzhou Medical University. Written informed consent was provided by the patients’ legal guardians.

## Author Contributions

SL, Z-GL, and W-PL designed the experiments. JuW, J-XL, X-GY, XL, Q-XZ, X-RL, JiW, L-DG, F-LL, B-ML, Z-FG, Q-HG, and Y-HY collected and analyzed the clinical data and patient samples. Z-LY and HL performed the computational modeling. SL, Z-GL, and W-PL wrote the manuscript, with contributions from all the authors.

## Conflict of Interest

The authors declare that the research was conducted in the absence of any commercial or financial relationships that could be construed as a potential conflict of interest.

## Publisher’s Note

All claims expressed in this article are solely those of the authors and do not necessarily represent those of their affiliated organizations, or those of the publisher, the editors and the reviewers. Any product that may be evaluated in this article, or claim that may be made by its manufacturer, is not guaranteed or endorsed by the publisher.

## Footnotes

1
gnomad.broadinstitute.org

2
https://epi25.broadinstitute.org/

3
https://zhanglab.ccmb.med.umich.edu/I-TASSER/

4
https://folding.biofold.org/i-mutant/i-mutant2.0.html

5
http://www.informatics.jax.org/allele/MGI:1934917

6
www.omim.org

## References

[B1] Aldinger K. A. Mosca S. J. Tetreault M. Dempsey J. C. Ishak G. E. Hartley T. (2014). Mutations in LAMA1 cause cerebellar dysplasia and cysts with and without retinal dystrophy. *Am. J. Hum. Genet.* 95 227–234. 10.1016/j.ajhg.2014.07.007 25105227 PMC4129402

[B2] Barak T. Kwan K. Y. Louvi A. Demirbilek V. Saygi S. Tuysuz B. (2011). Recessive LAMC3 mutations cause malformations of occipital cortical development. *Nat. Genet.* 43 590–594. 10.1038/ng.836 21572413 PMC3329933

[B3] Bayat A. Bayat M. Rubboli G. Moller R. S. (2021). Epilepsy syndromes in the first year of life and usefulness of genetic testing for precision therapy. *Genes (Basel)* 12:1051. 10.3390/genes12071051 34356067 PMC8307222

[B4] Capriotti E. Fariselli P. Casadio R. (2005). I-Mutant2.0: predicting stability changes upon mutation from the protein sequence or structure. *Nucleic Acids Res.* 33 W306–W310. 10.1093/nar/gki375 15980478 PMC1160136

[B5] Carvalho D. R. Giuliani L. R. Simao G. N. Santos A. C. Pina-Neto J. M. (2006). Autosomal dominant atretic cephalocele with phenotype variability: report of a Brazilian family with six affected in four generations. *Am. J. Med. Genet. A* 140 1458–1462. 10.1002/ajmg.a.31255 16718686

[B6] Chan S. H. Foley A. R. Phadke R. Mathew A. A. Pitt M. Sewry C. (2014). Limb girdle muscular dystrophy due to LAMA2 mutations: diagnostic difficulties due to associated peripheral neuropathy. *Neuromuscul. Disord.* 24 677–683. 10.1016/j.nmd.2014.05.008 24957499

[B7] GTEx Consortium (2013). The genotype-tissue expression (GTEx) project. *Nat. Genet.* 45 580–585. 10.1038/ng.2653 23715323 PMC4010069

[B8] CONVERGE Consortium (2015). Sparse whole-genome sequencing identifies two loci for major depressive disorder. *Nature* 523 588–591. 10.1038/nature14659 26176920 PMC4522619

[B9] Copp A. J. Carvalho R. Wallace A. Sorokin L. Sasaki T. Greene N. D. (2011). Regional differences in the expression of laminin isoforms during mouse neural tube development. *Matrix Biol.* 30 301–309. 10.1016/j.matbio.2011.04.001 21524702 PMC3565558

[B10] Darbro B. W. Mahajan V. B. Gakhar L. Skeie J. M. Campbell E. Wu S. (2013). Mutations in extracellular matrix genes NID1 and LAMC1 cause autosomal dominant Dandy-Walker malformation and occipital cephaloceles. *Hum. Mutat.* 34 1075–1079. 10.1002/humu.22351 23674478 PMC3714376

[B11] Domogatskaya A. Rodin S. Tryggvason K. (2012). Functional diversity of laminins. *Annu. Rev. Cell Dev. Biol.* 28 523–553. 10.1146/annurev-cellbio-101011-155750 23057746

[B12] Durkin M. E. Loechel F. Mattei M. G. Gilpin B. J. Albrechtsen R. Wewer U. M. (1997). Tissue-specific expression of the human laminin alpha5-chain, and mapping of the gene to human chromosome 20q13.2-13.3 and to distal mouse chromosome 2 near the locus for the ragged (Ra) mutation. *FEBS Lett.* 411 296–300. 10.1016/s0014-5793(97)00686-8 9271224

[B13] Elmas M. Gogus B. Solak M. (2020). Understanding what you have found: a family with a mutation in the LAMA1 gene with literature review. *Clin. Med. Insights Case Rep.* 13:1179547620948666. 10.1177/1179547620948666 32884387 PMC7440728

[B14] Eltze C. M. Chong W. K. Cox T. Whitney A. Cortina-Borja M. Chin R. F. (2013). A population-based study of newly diagnosed epilepsy in infants. *Epilepsia* 54 437–445. 10.1111/epi.12046 23252366

[B15] Epi25 Collaborative (2019). Ultra-rare genetic variation in the epilepsies: a whole-exome sequencing study of 17,606 individuals. *Am. J. Hum. Genet.* 105 267–282. 10.1016/j.ajhg.2019.05.020 31327507 PMC6698801

[B16] Gan J. Cai Q. Galer P. Ma D. Chen X. Huang J. (2019). Mapping the knowledge structure and trends of epilepsy genetics over the past decade: a co-word analysis based on medical subject headings terms. *Medicine (Baltimore)* 98:e16782. 10.1097/MD.0000000000016782 31393404 PMC6709143

[B17] Han J. Y. Jang J. H. Park J. Lee I. G. (2018). Targeted next-generation sequencing of korean patients with developmental delay and/or intellectual disability. *Front. Pediatr.* 6:391. 10.3389/fped.2018.00391 30631761 PMC6315160

[B18] Hirose G. (2013). [An overview of epilepsy: its history, classification, pathophysiology and management]. *Brain Nerve* 65 509–520. 23667116

[B19] Hohenester E. (2019). Structural biology of laminins. *Essays Biochem.* 63 285–295. 10.1042/EBC20180075 31092689 PMC6744579

[B20] Kang K. W. Kim W. Cho Y. W. Lee S. K. Jung K. Y. Shin W. (2019). Genetic characteristics of non-familial epilepsy. *PeerJ* 7:e8278. 10.7717/peerj.8278 31875159 PMC6925949

[B21] Karczewski K. J. Francioli L. C. Tiao G. Cummings B. B. Alfoldi J. Wang Q. (2020). The mutational constraint spectrum quantified from variation in 141,456 humans. *Nature* 581 434–443. 10.1038/s41586-020-2308-7 32461654 PMC7334197

[B22] Kikkawa Y. Miner J. H. (2006). Molecular dissection of laminin alpha 5 in vivo reveals separable domain-specific roles in embryonic development and kidney function. *Dev. Biol.* 296 265–277. 10.1016/j.ydbio.2006.04.463 16750824

[B23] Kivirikko S. McGrath J. A. Baudoin C. Aberdam D. Ciatti S. Dunnill M. G. (1995). A homozygous nonsense mutation in the alpha 3 chain gene of laminin 5 (LAMA3) in lethal (Herlitz) junctional epidermolysis bullosa. *Hum. Mol. Genet.* 4 959–962. 10.1093/hmg/4.5.959 7633458

[B24] Knoll R. Postel R. Wang J. Kratzner R. Hennecke G. Vacaru A. M. (2007). Laminin-alpha4 and integrin-linked kinase mutations cause human cardiomyopathy via simultaneous defects in cardiomyocytes and endothelial cells. *Circulation* 116 515–525. 10.1161/CIRCULATIONAHA.107.689984 17646580

[B25] Liang S. Crutcher K. A. (1992). Neuronal migration on laminin in vitro. *Brain Res. Dev. Brain Res.* 66 127–132. 10.1016/0165-3806(92)90148-p 1600626

[B26] Luckenbill-Edds L. (1997). Laminin and the mechanism of neuronal outgrowth. *Brain Res. Brain Res. Rev.* 23 1–27. 10.1016/s0165-0173(96)00013-6 9063584

[B27] Martin H. C. Jones W. D. McIntyre R. Sanchez-Andrade G. Sanderson M. Stephenson J. D. (2018). Quantifying the contribution of recessive coding variation to developmental disorders. *Science* 362 1161–1164. 10.1126/science.aar6731 30409806 PMC6726470

[B28] Maselli R. A. Arredondo J. Vazquez J. Chong J. X. Bamshad M. J. Nickerson D. A. (2018). A presynaptic congenital myasthenic syndrome attributed to a homozygous sequence variant in LAMA5. *Ann. N Y Acad. Sci.* 1413 119–125. 10.1111/nyas.13585 29377152 PMC6252105

[B29] McGrath J. A. Kivirikko S. Ciatti S. Moss C. Dunnill G. S. Eady R. A. (1995). A homozygous nonsense mutation in the alpha 3 chain gene of laminin 5 (LAMA3) in Herlitz junctional epidermolysis bullosa: prenatal exclusion in a fetus at risk. *Genomics* 29 282–284. 10.1006/geno.1995.1246 8530087

[B30] McLean W. H. Irvine A. D. Hamill K. J. Whittock N. V. Coleman-Campbell C. M. Mellerio J. E. (2003). An unusual N-terminal deletion of the laminin alpha3a isoform leads to the chronic granulation tissue disorder laryngo-onycho-cutaneous syndrome. *Hum. Mol. Genet.* 12 2395–2409. 10.1093/hmg/ddg234 12915477

[B31] Miner J. H. Cunningham J. Sanes J. R. (1998). Roles for laminin in embryogenesis: exencephaly, syndactyly, and placentopathy in mice lacking the laminin alpha5 chain. *J. Cell Biol.* 143 1713–1723. 10.1083/jcb.143.6.1713 9852162 PMC2132973

[B32] Mukherjee C. Saleem S. Das S. Biswas S. C. Bhattacharyya D. (2020). Human placental laminin: role in neuronal differentiation, cell adhesion and proliferation. *J. Biosci.* 45:93. 32713856

[B33] Nakano A. Chao S. C. Pulkkinen L. Murrell D. Bruckner-Tuderman L. Pfendner E. (2002). Laminin 5 mutations in junctional epidermolysis bullosa: molecular basis of Herlitz vs. non-Herlitz phenotypes. *Hum. Genet.* 110 41–51. 10.1007/s00439-001-0630-1 11810295

[B34] Omar M. H. Kerrisk Campbell M. Xiao X. Zhong Q. Brunken W. J. Miner J. H. (2017). CNS neurons deposit laminin alpha5 to stabilize synapses. *Cell Rep.* 21 1281–1292. 10.1016/j.celrep.2017.10.028 29091766 PMC5776391

[B35] Pierson M. Cordier J. Hervouuet F. Rauber G. (1963). [An unusual congenital and familial congenital malformative combination involving the eye and kidney]. *J. Genet. Hum.* 12 184–213. 14136829

[B36] Radmanesh F. Caglayan A. O. Silhavy J. L. Yilmaz C. Cantagrel V. Omar T. (2013). Mutations in LAMB1 cause cobblestone brain malformation without muscular or ocular abnormalities. *Am. J. Hum. Genet.* 92 468–474. 10.1016/j.ajhg.2013.02.005 23472759 PMC3591846

[B37] Salvati A. Bonaventura E. Sesso G. Pasquariello R. Sicca F. (2021). Epilepsy in LAMA2-related muscular dystrophy: a systematic review of the literature. *Seizure* 91 425–436. 10.1016/j.seizure.2021.07.020 34325301

[B38] Sampaolo S. Napolitano F. Tirozzi A. Reccia M. G. Lombardi L. Farina O. (2017). Identification of the first dominant mutation of LAMA5 gene causing a complex multisystem syndrome due to dysfunction of the extracellular matrix. *J. Med. Genet.* 54 710–720. 10.1136/jmedgenet-2017-104555 28735299

[B39] Song T. Y. Deng J. Fang F. Chen C. H. Wang X. H. Wang X. (2021). [The etiology of 340 infants with early-onset epilepsy]. *Zhonghua Er Ke Za Zhi* 59 387–392. 10.3760/cma.j.cn112140-20201016-00947 33902223

[B40] Tonduti D. Dorboz I. Renaldo F. Masliah-Planchon J. Elmaleh-Berges M. Dalens H. (2015). Cystic leukoencephalopathy with cortical dysplasia related to LAMB1 mutations. *Neurology* 84 2195–2197. 10.1212/WNL.0000000000001607 25925986

[B41] Vidal F. Baudoin C. Miquel C. Galliano M. F. Christiano A. M. Uitto J. (1995). Cloning of the laminin alpha 3 chain gene (LAMA3) and identification of a homozygous deletion in a patient with Herlitz junctional epidermolysis bullosa. *Genomics* 30 273–280. 10.1006/geno.1995.9877 8586427

[B42] Wang J. Lin Z. J. Liu L. Xu H. Q. Shi Y. W. Yi Y. H. (2017). Epilepsy-associated genes. *Seizure* 44 11–20. 10.1016/j.seizure.2016.11.030 28007376

[B43] Wang J. Y. Zhou P. Wang J. Tang B. Su T. Liu X. R. (2018). ARHGEF9 mutations in epileptic encephalopathy/intellectual disability: toward understanding the mechanism underlying phenotypic variation. *Neurogenetics* 19 9–16. 10.1007/s10048-017-0528-2 29130122

[B44] Wirrell E. C. Grossardt B. R. Wong-Kisiel L. C. Nickels K. C. (2011). Incidence and classification of new-onset epilepsy and epilepsy syndromes in children in Olmsted County, Minnesota from 1980 to 2004: a population-based study. *Epilepsy Res.* 95 110–118. 10.1016/j.eplepsyres.2011.03.009 21482075 PMC3260338

[B45] Wiszniewski W. Gawlinski P. Gambin T. Bekiesinska-Figatowska M. Obersztyn E. Antczak-Marach D. (2018). Comprehensive genomic analysis of patients with disorders of cerebral cortical development. *Eur. J. Hum. Genet.* 26 1121–1131. 10.1038/s41431-018-0137-z 29706646 PMC6057976

[B46] Xiong H. Tan D. Wang S. Song S. Yang H. Gao K. (2015). Genotype/phenotype analysis in Chinese laminin-alpha2 deficient congenital muscular dystrophy patients. *Clin. Genet.* 87 233–243. 10.1111/cge.12366 24611677

[B47] Yang J. Zhang Y. (2015). I-TASSER server: new development for protein structure and function predictions. *Nucleic Acids Res.* 43 W174–W181. 10.1093/nar/gkv342 25883148 PMC4489253

[B48] Zenker M. Tralau T. Lennert T. Pitz S. Mark K. Madlon H. (2004). Congenital nephrosis, mesangial sclerosis, and distinct eye abnormalities with microcoria: an autosomal recessive syndrome. *Am. J. Med. Genet. A* 130A 138–145. 10.1002/ajmg.a.30310 15372515

