# Supplementary material for: Recessive LAMA5 Variants Associated With Partial Epilepsy and Spasms in Infancy
Source: Front Mol Neurosci. 2022 May 19;15:825390. doi: 10.3389/fnmol.2022.825390 (PMC9162154; doi:10.3389/fnmol.2022.825390)
Supplement: Supplementary file 1 [file Table_1.DOCX]

**Table S1. Genetic Features of the *LAMA5* Mutations**

|  | **cDNA change (NM_014689.2)** | **Protein change** | **Inheritance** | **MAF** | **SIFT** | **PP2-HDIV** | **Mutation-Taster** | **CADD** | **Geno-Canyon** | **fitCons** | **M_CAP** | **GERP** |
| --- | --- | --- | --- | --- | --- | --- | --- | --- | --- | --- | --- | --- |
| **Case 1** | c.1337G>A | p.Arg446Gln | Paternal | 2.14×10^-5^ | T (0.149) | D (1) | D (1) | D (25.3) | D (1) | D (0.707) | D (0.085) | C (4.78) |
|  | c.10699C>T | p.Pro3567Ser | Maternal | 5.48×10^-5^ | T (0.304) | B (0.27) | P (1) | T (0.201) | D (1) | T (0.672) | D (0.068) | NC (1.99) |
| **Case 2** | c.1418G>A | p.Pro473Leu | Paternal | 5.69×10^-5^ | D (0.012) | D (0.939) | D (1) | D (22.5) | D (1) | D (0.707) | D (0.063) | C (3.52) |
|  | c.3608C>T | p.Arg1203Gln | Maternal | 1.04×10^-4^ | T (0.691) | D (0.73) | P (1) | T (14.87) | T (0.726) | D (0.707) | T (0.015) | NC (0.962) |
| **Case 3** | c.5426C>T | p.Arg1809His | Paternal | 3.98×10^-6^ | T (0.2) | B (0.008) | D (0.941) | T (13.08) | T (0.998) | D (0.707) | D (0.03) | NC (-1.25) |
|  | c.7394C>T | p.Arg2465Gln | Maternal | 4.26×10^-4^ | T (0.069) | B (0.44) | P (0.999) | D (20.8) | D (1) | D (0.707) | D (0.034) | C (3.54) |
| **Case 4** | c.3170C>T | p.Ser1057Leu | Paternal | NA | D (0.041) | D (0.938) | P (1) | D (23.4) | D (1) | D (0.707) | D (0.036) | C (3.92) |
|  | c.6388C>T | p.Arg2130Cys | Maternal | 4.78×10^-4^ | D (0.001) | D (0.974) | D (0.510) | D (24.0) | D (1) | T (0.672) | D (0.034) | C (3.28) |
| **Case 5** | c.9448G>A | p.Gly3150Ser | Paternal | 3.96×10^-4^ | T (0.544) | D (0.991) | D (1) | T (19.3) | D (1) | D (0.707) | T (0.021) | C (4.06) |
|  | c.10744C>T | p.Arg3582Trp | Maternal | 2.13×10^-4^ | T (0.184) | B (0.023) | P (1) | D (24.9) | D (1) | T (0.672) | D (0.1) | NC(-0.529) |
| **Case 6** | c.1963G>A | p.Gly655Ser | Maternal | 2.82×10^-5^ | D (0.04) | D (0.728) | P (0.827) | D (24) | D (1) | D (0.707) | D (0.115) | C (2.18) |
|  | c.2192C>G | p.Ala731Gly | Paternal | 4.98×10^-5^ | D (0.016) | D (0.999) | D (0.779) | D (23.2) | D (1) | T (0.672) | T (0.022) | C (2.98) |

**Abbreviations:** B, benign; C, conserved; CADD, combined annotation dependent depletion; D, damaging; DC, disease causing; fitCons, fitness consequences of functional annotation; GERP, Genomic Evolutionary Rate Profiling; MAF, minor allele frequency from Genome Aggregation Database; M-CAP, Mendelian Clinically Applicable Pathogenicity; NA: not applicable; PD, probably damaging; T, tolerable. SIFT, Sorting Intolerant From Tolerant
